# Rett Syndrome Mutant Neural Cells Lacks MeCP2 Immunoreactive Bands

**DOI:** 10.1371/journal.pone.0153262

**Published:** 2016-04-11

**Authors:** Carlos Bueno, Rafael Tabares-Seisdedos, Jose M. Moraleda, Salvador Martinez

**Affiliations:** 1 IMIB-Arrixaca and Faculty of Medicine, University of Murcia, Murcia and CIBERSAM, Murcia, Spain; 2 Cell Therapy Unit, Hospital Universitary Virgen de la Arrixaca, IMIB-Arrixaca and Faculty of Medicine, University of Murcia, Murcia, Spain; 3 Teaching Unit of Psychiatry and Psychological Medicine, Department of Medicine, University of Valencia and CIBERSAM, Valencia, Spain; 4 Instituto de Neurociencias UMH-CSIC, 03550, San Juan de Alicante, Univ, Miguel Hernandez, Spain; Institute of Genetics and Biophysics, ITALY

## Abstract

Dysfunctions of MeCP2 protein lead to various neurological disorders such as Rett syndrome and Autism. The exact functions of MeCP2 protein is still far from clear. At a molecular level, there exist contradictory data. MeCP2 protein is considered a single immunoreactive band around 75 kDa by western-blot analysis but several reports have revealed the existence of multiple MeCP2 immunoreactive bands above and below the level where MeCP2 is expected. MeCP2 immunoreactive bands have been interpreted in different ways. Some researchers suggest that multiple MeCP2 immunoreactive bands are unidentified proteins that cross-react with the MeCP2 antibody or degradation product of MeCP2, while others suggest that MeCP2 post-transcriptional processing generates multiple molecular forms linked to cell signaling, but so far they have not been properly analyzed in relation to Rett syndrome experimental models. The purpose of this study is to advance understanding of multiple MeCP2 immunoreactive bands in control neural cells and p.T158M MeCP2e1 mutant cells. We have generated stable wild-type and p.T158M MeCP2e1-RFP mutant expressing cells. Application of N- and C- terminal MeCP2 antibodies, and also, RFP antibody minimized concerns about nonspecific cross-reactivity, since they react with the same antigen at different epitopes. We report the existence of multiple MeCP2 immunoreactive bands in control cells, stable wild-type and p.T158M MeCP2e1-RFP mutant expressing cells. Also, MeCP2 immunoreactive bands differences were found between wild-type and p.T158M MeCP2e1-RFP mutant expressing cells. Slower migration phosphorylated band around 70kDa disappeared in p.T158M MeCP2e1-RFP mutant expressing cells. These data suggest that threonine 158 could represent an important phosphorylation site potentially involved in protein function. Our results clearly indicate that MeCP2 antibodies have no cross-reactivity with similar epitopes on others proteins, supporting the idea that MeCP2 may exist in multiple different molecular forms and that molecular pattern variations derived from altered post-transcriptional processing may underlay Rett syndrome physiophatology

## Introduction

Methyl-CpG-binding protein 2 (MeCP2) was initially identified in 1992 as a classic methyl-CpG-binding protein [1]. The knowledge about MeCP2 protein function has changed over time, from being considered a single function protein [2] to a multifunctional nuclear protein [3]. Dysfunctions of human MeCP2 protein (hMeCP2) lead to various neurological disorders [4] such as Rett syndrome [5] and Autism [6]. In human and mouse, MeCP2 exists in two different isoforms produced by alternative splicing differing at the N-termini due to exclusion or inclusion of exon 2 respectively. Conventional western-blot analysis would not allow resolution of the two MeCP2 isoforms [7,8].

The exact functions of MeCP2 protein is still far from clear. At a molecular level, there exist contradictory data. MeCP2 protein is considered a single MeCP2 immunoreactive band around 75 kDa by western-blot analysis [9] but several reports have revealed the existence of multiple MeCP2 immunoreactive bands above and below the level where MeCP2 is expected. Higher molecular weight form of hMeCP2 has been reported to be expressed in human frontal cortex nuclear and synaptic fractions and in lymphoid cells as well [10]. Lower molecular weight form of MeCP2 has been reported in rat brain nuclear extract [1,11], wild-type and mutant mouse brain [12–15] and MeCP2 transfected cells [16]. Higher and lower molecular weight form of hMeCP2 has been reported to be expressed in fibroblast and lymphoblastoid strains from females with clinically diagnosed Rett syndrome [17] and MeCP2 transfected cells [18].

Multiple MeCP2 immunoreactive bands have been interpreted in different ways. Some researchers suggest that multiple MeCP2 immunoreactive bands are unidentified proteins that cross-react with the MeCP2 antibody [11,12,15–17] or degradation product of MeCP2 [1,14], while others suggest that hMeCP2 post-transcriptional processing generates multiple molecular forms linked to cell signaling [10,18].

In addition, many MeCP2 antibodies available commercially against different epitopes of MeCP2 protein detected multiple bands by western-blot analysis (Table 1).

**Table 1:** MeCP2 antibodies available commercially against different epitopes of MeCP2 protein detected multiple bands by western-blot analysis.

Name of antibody	Comercial supplier	MeCP2 Immunogen	Species isotype	Aproximate sizes (KDa)
Ab137358	Abcam	a.a.1-232	Rabbit IgG	95,70
Ab117567	Abcam	a.a.1-486	Mouse IgG	95,75,50,40
Ab50005	Abcam	a.a.471-486	Mouse IgG	75,50
Ab2829	Abcam	a.a.469-486	Rabbit IgG	55,40
Ab119955	Abcam	a.a.2-211	Rabbit IgG	55,34
Ab75716	Abcam	a.a. surrounding S421	Rabbit IgG	75,55,40
SAB2101453	Sigma-Aldrich	a.a.36-85	Rabbit IgG	95,40
HPA000593	Sigma-Aldrich	a.a.239-350	Rabbit IgG	50,70,30
WH0004204M1	Sigma-Aldrich	a.a.81-170	Mouse IgG	75,50
OAAB15948	Avivasybio	a.a.3-33	Rabbit IgG	95,75,50,40
ARP32694P050	Avivasybio	a.a.35-84	Rabbit IgG	95,45
OAPC00174	Avivasybio	a.a. surrounding S80	Rabbit IgG	75,50
H00004204-M01	Abnova	a.a.81-170	Mouse IgG	95,75,50,40
H00004204-D01P	Abnova	a.a.1-486	Rabbit IgG	75,50
SC-137070	Santa Cruz	a.a.185-486	Mouse IgG	90,55
SC-5755	Santa Cruz	N-terminus	Rabbit IgG	90,75,55
PA5-12234	Thermofisher	a.a.400-408	Rabbit IgG	75,55,40
PA1-887	Thermofisher	a.a.1-15	Rabbit IgG	55,40
ABE28	Merck-Millipore	a.a.1-486/K464-ac	Rabbit IgG	110,80,50
ABE171	Merck-Millipore	a.a.1-486	Chicken IgY	100,80,70,40

The purpose of this study is to advance understanding of MeCP2 multiple immunoreactive bands in wild*-*type and MeCP2 mutant neural cells. In the present study, we found that immunoblots of whole several control neural cell lines lysates revealed the existence of multiple MeCP2 immunoreactive bands above and below the level where MeCP2 is expected.

To test the specificity of MeCP2 antibodies, we have generated stable wild-type and p.T158M MeCP2e1-red fluorescence protein (RFP) fusion protein mutant expressing neural cells. Application of N- and C- terminal MeCP2 antibodies, and also, RFP antibody minimized concerns about nonspecific cross-reactivity, since they react with the same antigen at different epitopes.

No large differences in the apparent molecular weight (MWa) of MeCP2 immunoreactive bands were noticed between control neural cells and hMeCP2e1-RFP stable transfected neural cell lines. In addition, staining with RFP antibody, that minimized concerns about nonspecific cross-reactivity, produced blots with similar pattern. Futhermore, no large differences in the apparent molecular weight of MeCP2 immunoreactive bands were noticed between our results, previous reports and MeCP2 antibodies available commercially against different epitopes of MeCP2 protein.

To demonstrate the specificity of multiple MeCP2 immunoreactive bands detected in hMeCP2e1-RFP expressing neural cell lines, and therefore, definitely exclude the cross-reactivity with similar epitopes on other proteins, we performed MeCP2e1-RFP^+^ protein detection via SDS-PAGE and in-gel fluorescence scanning. After the fluorescence scan, proteins in gels were transferred to nitrocellulose membranes for western blotting. The immunoblot with antibody against MeCP2 revealed multiple MeCP2 immunoreactive bands at the same position as the fluorescent signals.

Lastly, MeCP2 immunoreactive bands differences were found between stable wild-type and p.T158M MeCP2e1-RFP mutant expressing neural cells. Slower migration phosphorylated band around 70kDa disappeared in p.T158M MeCP2e1-RFP mutant expressing cells. These data suggest that threonine 158 could represent an important phosphorylation site potentially involved in protein function.

Our results clearly indicate that MeCP2 antibodies have no cross-reactivity with similar epitopes on others proteins, supporting the idea that MeCP2 may exist in multiple different molecular forms and that molecular pattern variations derived from altered post-transcriptional processing may underlay Rett syndrome physiophatology

## Materials and Methods

### Cell Culture

Human embryonic kidney HEK293 (ATCC No. CRL-1573) cell line, human neuroblastoma SH-SY5Y (ATCC No. CRL-2266) cell line and murine neuroblastoma Neuro-2A (N2A; ATCC No. CCL-131) cell line were maintained in a growth medium Dulbecco’s Modified Eagle’s Medium (DMEM) supplemented with 10% fetal bovine serum, 100 units/ml penicillin-streptomycin and 2 mM L-glutamine. Rat pheochromocytoma PC12 (ATCC No. CRL-1721) cell line was maintained in a growth medium (DMEM) supplemented with 5% fetal bovine serum, 10% horse serum, 100 units/ml penicillin-streptomycin and 2 mM L-glutamine. The cell lines were incubated at 37°C in 5% CO2. All cell cultured reagents were from Sigma-Aldrich (St. Louis, MO, USA).

### Generation of wild type and p.T158M hMeCP2e1-mRFP mutant fusion proteins

We used human cDNA clones (Genebank:BQ072357 and Genebank:BC011612.1) as template to generate full lenght hMeCP2e1 coding sequence. The PCR products were inserted into pSTBlue1 vector (Millipore, Billerica, MA, USA). hMeCP2e1 coding region without stop codon was subcloned into the pSTBlue1-mRFP vector to obtain hMeCP2e1-RFP in-frame fusion protein. Mutant hMeCP2e1-RFP (p.T158M) was generated using QuickChange II site-directed mutagenesis Kit^®^ (Angilent Technologies, Santa Clara, CA, USA). We used the pSTBlue1-hMeCP2e1-RFP as template and the following primers:

hMeCP2e1T158M/se 5'GACCCTAATGATTTTGACTTCATGGTAACTGGGAGAGGGAGCCCC3'.

hMeCP2e1T158M/as 5'GGGCTCCCTCTCCCAGTTACCATGAAGTCAAAATCATTAGGGTCC3'.

Wild-type and mutant hMeCP2e1-RFP fusion protein were subcloned into pIRES1hyg bicistronic expression vector (Clontech, Cambridge, UK). DNA fragments were identified by restriction enzyme analysis and confirmed by double-stranded DNA sequencing.

### Transfection methods

One day before transfection the cells were seeded at a density of 0.5x10^5^ cells/cm2 in multi-well (12 or 24-well) plates. The cells were incubated with Lipofectamine 2000 (Invitrogen, Carlsbad, CA, USA), for 4 hours (following the supplier’s instructions), after which the lipofection mix was removed and replaced with fresh medium. Drug selection of stable transfectants was performed with 50–100 mg/ml hygromycin B (hyg; Calbiochem, La Jolla, CA, USA).

### Western Blotting

Cell lines were harvested using trypsin/EDTA (Gibco), washed twice with PBS, resuspended in RIPA lysis buffer (Millipore, Temecula, CA, USA) for 30 min at 4°C in the presence of protease inhibitors (Pierce^™^ protease inhibitor Mini Tables, Pierce Biotechnology Inc, Illinois, USA), PMSF 1M (Abcam, Cambridge, UK) and in the presence-absence of phosphatase inhibitor (PhosSTOP, Sigma-Aldrich). Protein concentration was determined using the bradford protein assay (Sigma-Aldrich). Proteins were separated in a 8% SDS-polyacryamide gel (SDS-PAGE) and transferred to a nitrocellulose membrane (Whatman, Maidstone, Kent, UK). PageRuler^™^ Prestained Protein Ladder (Thermo Scientific, Grand Island, NY, USA) has been used as size standards in protein electrophoresis (SDS-PAGE) and western-blotting. After transfer, nitrocellulose membranes were stained with Ponceau S solution (Sigma-Aldrich) to visualize protein bands. Blots were then incubated over-night at 4°C with rabbit antibody against MeCP2 (H-300,1:1000, Santa Cruz, Santa Cruz, CA, USA), rabbit antibody against RFP (PM005; 1:1000, MBL International Corporation, Woburn, MA, USA), mouse antibody against MeCP2 (AAH11612, Sigma-Aldrich) and mouse antibody against β-actin (A5441, 1:10000; Sigma-Aldrich). Secondary antibodies were used at 1:7000 for peroxidase anti-mouse Ab (PI-2000, Vector Laboratories, Burlingame, CA, USA) and 1:5,000 for peroxidase anti-rabbit Ab (PI-1000, Vector Laboratories). Immunoreactivity was detected using the enhanced chemiluminescence (ECL) Western blot detection system (Amersham Biosciences Europe, Freiberg, Germany) and Luminata^™^ Forte (Millipore corporation) using ImageQuant LAS 500 Gel Documentation System (GE Healthcare, Little Chalfont, UK) and G:Box Gel Documentation System (Syngene, Cambridge, UK).

### Fluorescence analyses

Photography of fluorescent cells were carried out in an inverted Leica CTR 6000 microscope equipped with a digital camera Leica DC500 or Leica DM IRB microscope equipped with a digital camera Leica DFC350FX (Leica Microsystems, Wetzar, Germany). In-gel fluorescence scanning was performed on a Typhoon FLA 9500 scanner (GE Healthcare, Little Chalfont, UK) using 432 nm excitation laser and 610 BP40 emmision filter.

## Results

### Multiple MeCP2 immunoreactive bands in neural cells

**Fig 1:**
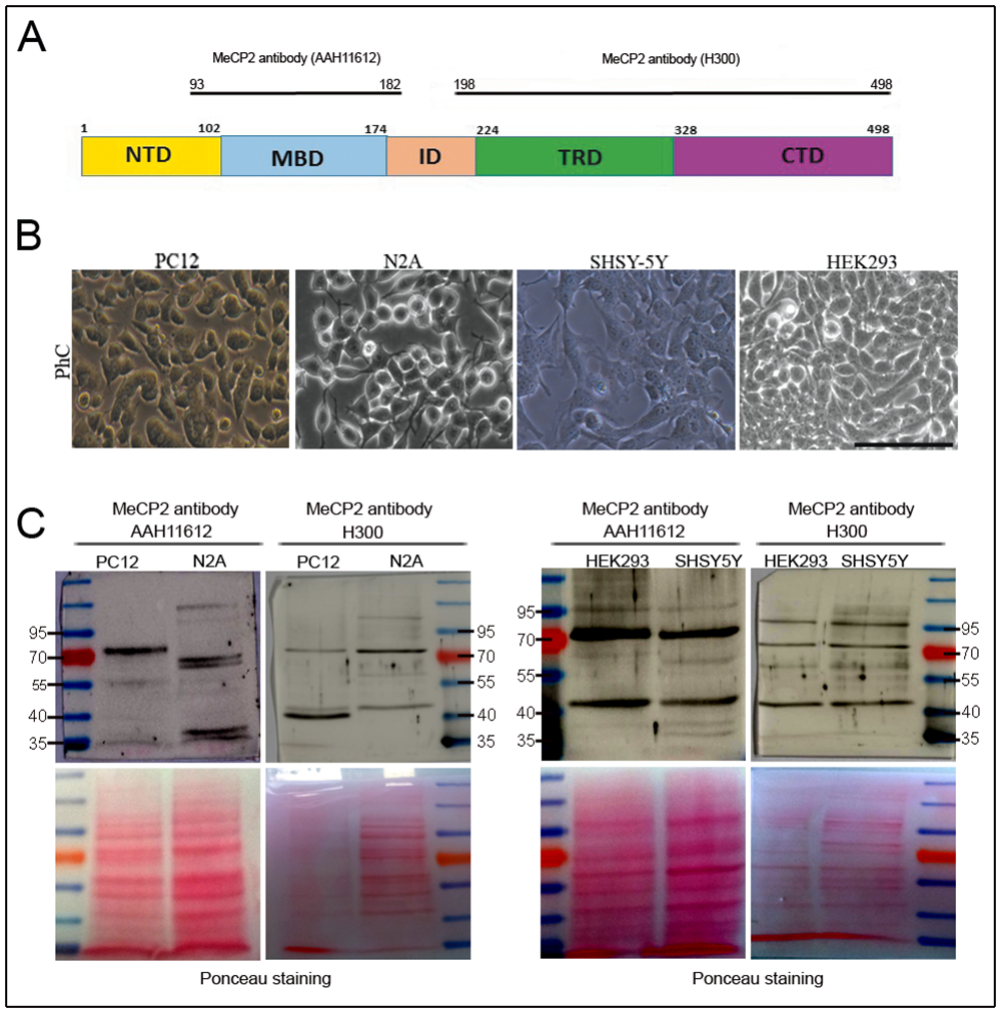
Multiple MeCP2 immunoreactive bands in neural cell lines. (**A**) Diagram of the hMeCP2e1 protein illustrating the position of the MeCP2 antibodies. (**B**) Phase-contrast photomicrographs (PhC) of proliferating neural cell lines. Scale bar = 100μm. (**C**) Western-blot analysis of proliferating neural cell lines with antibodies against the N-terminal (AAH11612, a.a.93-182) and C-terminal region (H300, a.a.198-496) of MeCP2 protein. Blots were stained with Ponceau solution as a loading control. Protein size markers (in kilodaltons) are indicated on the side of each panel.

As noted in the introduction, the purpose of this study is to advance understanding multiple MeCP2 immunoreactive bands above and below the level where MeCP2 is expected. To assess MeCP2 expression at the protein level, immunoblot analysis with antibodies against the N-terminal (AAH11612, a.a.93-182) and C-terminal region (H300, a.a.198-496) of hMeCP2 protein (Fig 1A) was carried out on total cell lysate from proliferating human and murine neural cell lines (Fig 1B). HEK293 cell line may be neuronal in origin [19].

Staining with the N-terminal MeCP2 antibody, the MWa of immunoreactive bands in PC12 cells was around 70 kDa, 55kDa and 35 kDa, while with C-terminal MeCP2 antibody, the MWa of immunoreactive bands was around 70kDa and 40kDa (two bands) (Fig 1C).

Staining with the N-terminal MeCP2 antibody, the MWa of immunoreactive bands in N2A cells was around 95 kDa, 70kDa (two bands), 55kDa and 35 kDa (two bands), while with C-terminal MeCP2 antibody, the MWa of immunoreactive bands was around 95kDa, 70kDa, 55kDa and 40kDa (Fig 1C).

Staining with the N-terminal MeCP2 antibody, the MWa of immunoreactive bands in HEK293 cells was around 95kda, 70kDa and 40kDa, while with C-terminal MeCP2 antibody, the MWa of immunoreactive bands was around 95kDa, 70kDa, 55kDa and 40kDa (Fig 1C).

Staining with the N-terminal MeCP2 antibody, the MWa of immunoreactive bands in SHSY5Y cells was around 70 kDa, 55kDa, 40kDa and 35kDa (two bands), while with C-terminal MeCP2 antibody, the MWa of immunoreactive bands was around 95kDa, 70kDa, 55kDa and 40kDa (Fig 1C).

### Multiple MeCP2 and RFP immunoreactive bands in hMeCP2e1-RFP expressing neural cells

**Fig 2:**
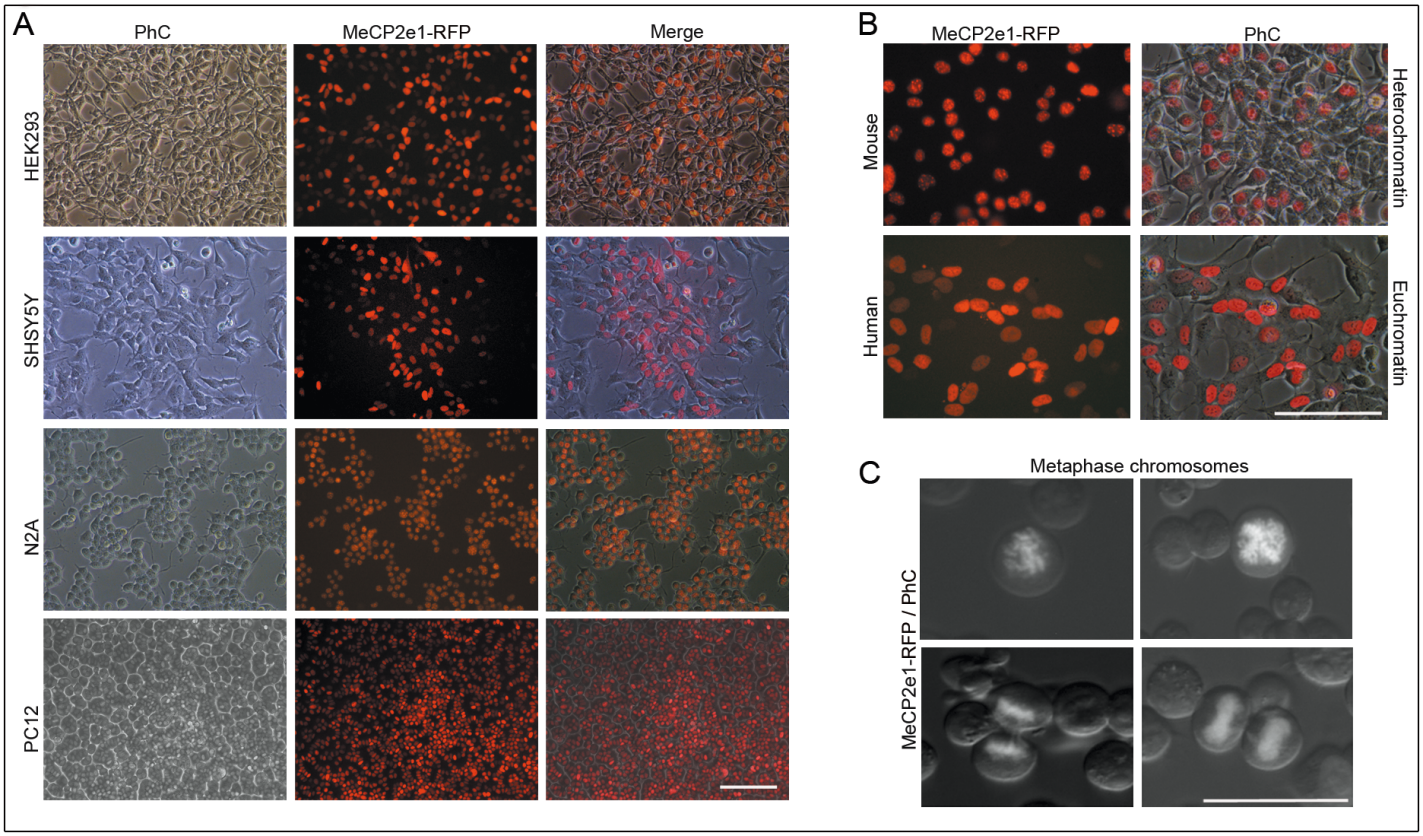
Correct localization of hMeCP2e1-RFP fusion protein in stable transfected neural cell lines. (**A**). Photomicrographs show phase-contrast (PhC) and fluorescence images of hMeCP2e1-RFP^+^ expressing neural cell lines. Scale bar = 100μm. (**B**) Nuclear localization of hMeCP2e1-RFP in mouse and human interphase nuclei. Scale bar = 100μm. (**C**) hMeCP2e1-RFP fusion protein localized to metaphase chromosomes in mitotic nuclei. Scale bar = 50μm.

To test the specificity of MeCP2 antibodies, we have generated hMeCP2e1-RFP expression vector (as described in Methods). This fusion protein can be detected with MeCP2 and RFP antibodies. Application of MeCP2 and RFP antibodies minimized concerns about nonspecific cross-reactivity, since they react with the same antigen at different epitopes. Neural cell lines were transfected by lipofection using the p(hMeCP2e1-RFP)IRES1hyg plasmid vector (as described in Methods). hMeCP2e1-RFP transfected cells, after months of continuous drug selection, rendered vigorously growing cultures in which most of cells were fluorescent under the microscope (Fig 2A). Previous immunofluorescence studies have shown strong localization of MeCP2 to methaphase chromosomes in mitotic nuclei [1] and also to pericentric heterochromatin in the mouse, whereas more diffuse staining is seen in human interphase nuclei [20]. hMeCP2e1-RFP fusion protein was correctly localized in proliferating neural cell lines (Fig 2B and 2C).

**Fig 3:**
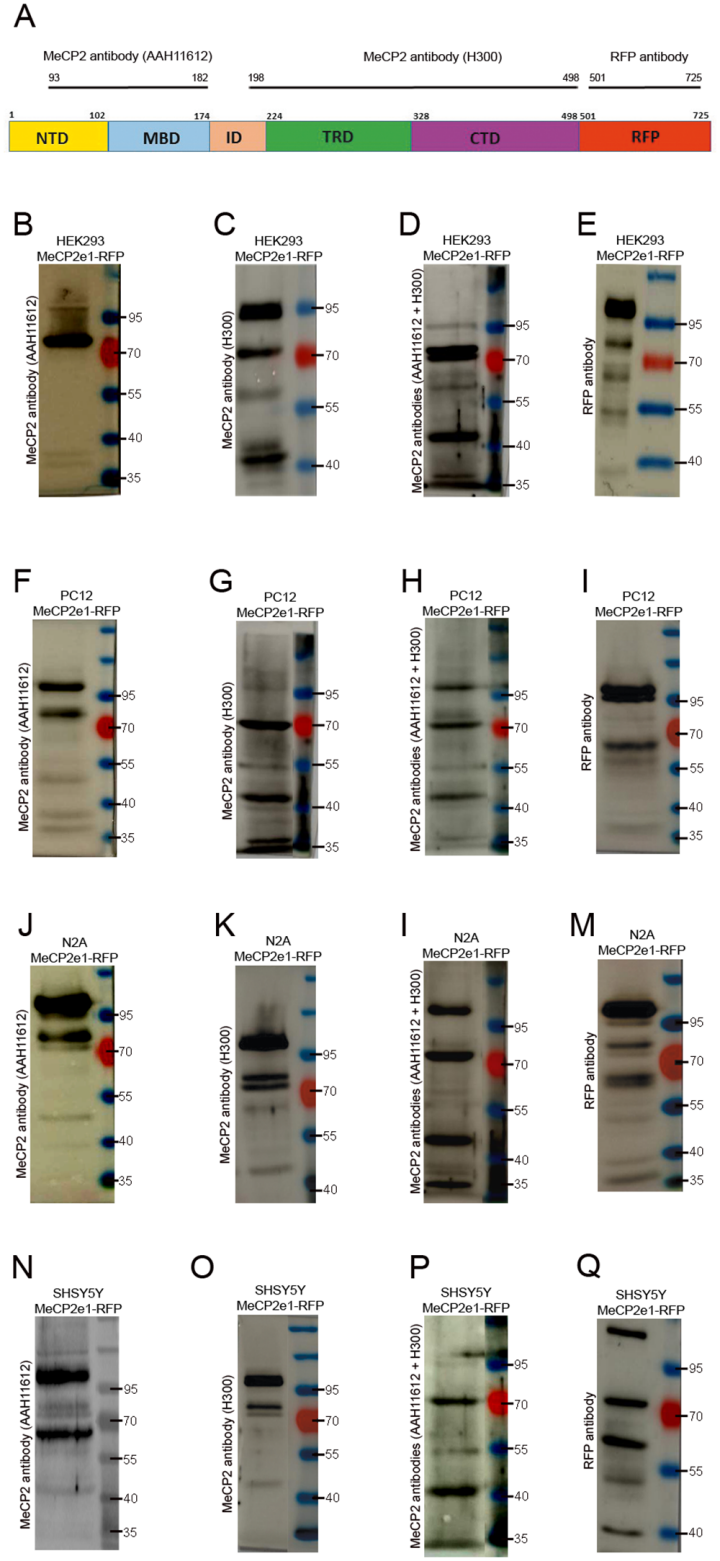
Multiple MeCP2 and RFP immunoreactive bands in hMeCP2e1-RFP expressing neural cell lines. (**A**) Diagram of the hMeCP2e1-RFP protein illustrating the position of the MeCP2 and RFP antibodies. (**B-D**) Western-blot analysis of hMeCP2e1-RFP^+^ HEK293 cell line with antibodies against the N- and C-terminal region of MeCP2. (**E**) RFP immunoreactive bands in transfected HEK293 cell line. (**F-H)** Western-blot analysis of hMeCP2e1-RFP^+^ PC12 cell line with antibodies against the N- and C-terminal region of MeCP2. **(I)** RFP immunoreactive bands in transfected PC12 cell line. **(J-L)** Western-blot analysis of hMeCP2e1-RFP^+^ N2A cell line with antibodies against the N- and C-terminal region of MeCP2. (**M**) RFP immunoreactive bands in transfected N2A cell line. (**N-P**) Western-blot analysis of hMeCP2e1-RFP^+^ SHSY5Y cell line with antibodies against the N- and C-terminal region of MeCP2. (**Q**) RFP immunoreactive bands in transfected SHSY5Y cell line. Protein size markers (in kilodaltons) are indicated on the right of each panel.

To assess MeCP2 expression at the protein level, immunoblot analysis with antibodies against the N-terminal (AAH11612, a.a.93-182) and C-terminal region (H300, a.a.198-496) of MeCP2 protein, and also, antibody against RFP (Fig 3A) was carried out on total cell lysate from proliferating hMeCP2e1-RFP expressing neural cell lines (Fig 3B–3Q).

Staining with the N-terminal MeCP2 antibody, the MWa of immunoreactive bands in hMeCP2e1-RFP^+^ HEK293 cells was around 95 kDa, 70 kDa and 35 kDa (two bands) (Fig 3B), while with C-terminal MeCP2 antibody, the MWa of immunoreactive bands was around 95kDa, 70kDa, 55kDa and 40kDa (two bands) (Fig 3C). Double staining with N- and C-terminal MeCP2 antibodies, the MWa of immunoreactive bands was around 95 kDa, 70 kDa (double band), 55 kDa, 40kDa and 35 kDa (Fig 3D), while with RFP antibody, the MWa of immunoreactive bands was around 95kDa, 70kDa (two bands), 55kDa and 40kDa (Fig 3E).

Staining with the N-terminal MeCP2 antibody, the MWa of immunoreactive bands in hMeCP2e1-RFP^+^ PC12 cells was around 95 kDa, 70 kDa, 55kDa and 35 kDa (two bands) (Fig 3F), while with C-terminal MeCP2 antibody, the MWa of immunoreactive bands was around 95kDa, 70kDa, 55kDa, 40kDa and 35kDa (Fig 3G). Double staining with N- and C-terminal MeCP2 antibodies, the MWa of immunoreactive bands was around was around 95 kDa, 70 kDa, 55 kDa, 40kDa and 35 kDa (Fig 3H), while with RFP antibody, the MWa of immunoreactive bands was around 95kDa (two bands), 70kDa (two bands), 55kDa and 35kDa (Fig 3I).

Staining with the N-terminal MeCP2 antibody, the MWa of immunoreactive bands in hMeCP2e1-RFP^+^ N2A cells was around 95 kDa, 70 kDa (two bands), 55 kDa and 40kDa (Fig 3J), while with C-terminal MeCP2 antibody, the MWa of immunoreactive bands was around 95kDa, 70kDa (three bands) and 40kDa (Fig 3K). Double staining with N- and C-terminal MeCP2 antibodies, the MWa of immunoreactive bands was around was around 95 kDa, 70 kDa, 55 kDa, 40kDa and 35 kDa (Fig 3L), while with RFP antibody, the MWa of immunoreactive bands was around 95kDa, 70kDa (two bands), 55kDa, 40kDa and 35kDa (Fig 3M).

Staining with the N-terminal MeCP2 antibody, the MWa of immunoreactive bands in hMeCP2e1-RFP^+^ SHSY5Y cells was around 95 kDa (doble band), 70 kDa (three bands), 55 kDa and 40kDa (Fig 3N), while with C-terminal MeCP2 antibody, the MWa of immunoreactive bands was around 95kDa, 70kDa (two bands) and 40kDa (Fig 3O). Double staining with N- and C-terminal MeCP2 antibodies, the MWa of immunoreactive bands was around was around 95 kDa, 70 kDa, 55 kDa, 40kDa and 35 kDa (Fig 3P), while with RFP antibody, the MWa of immunoreactive bands was around 95kDa, 70kDa (two bands), 55kDa and 40kDa (Fig 3Q).

No large differences in the MWa of multiple MeCP2 immunoreactive bands were noticed between control cells and hMeCP2e1-RFP stable transfected neural cell lines although the intensity of MeCP2 and RFP immunoreactive bands sometimes varied from one experiment to another. Application of N- and C- terminal MeCP2 antibodies, and also, RFP antibody minimized concerns about nonspecific cross-reactivity, since they react with the same antigen at different epitopes.

**Fig 4:**
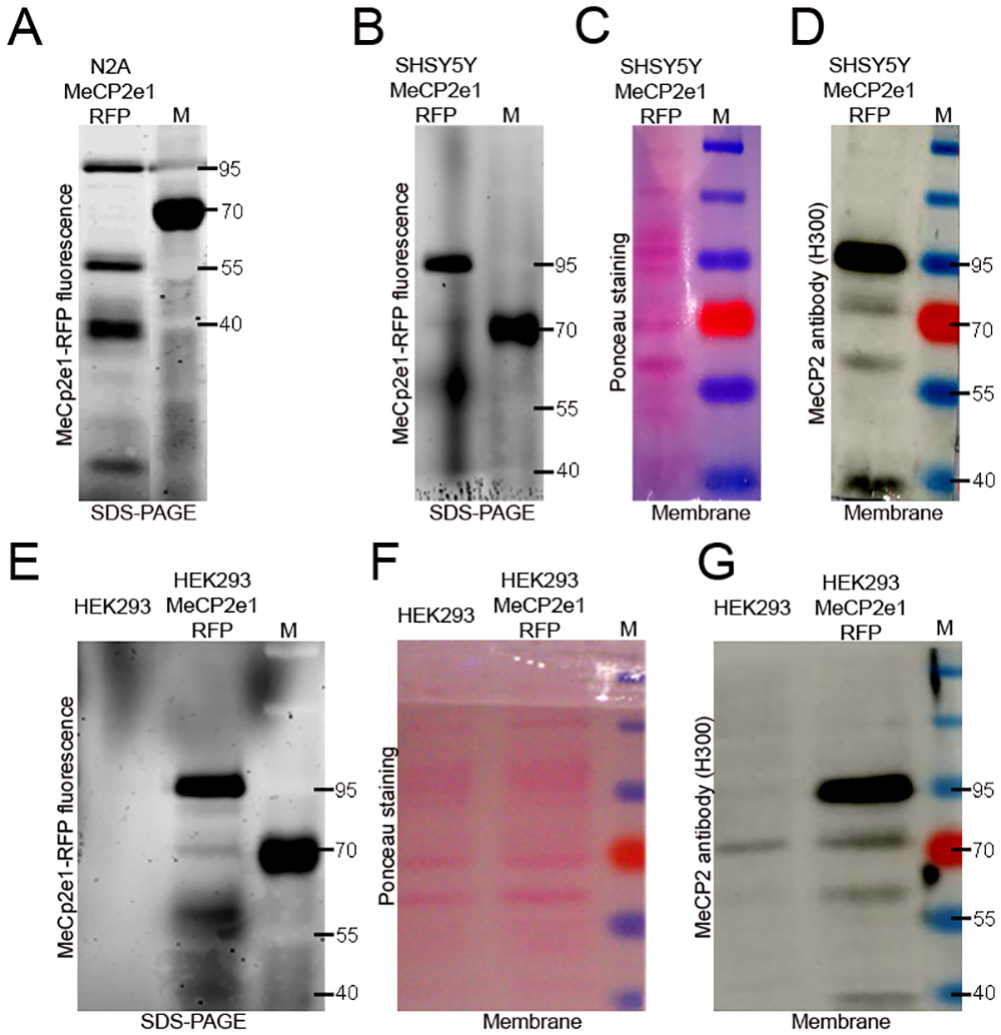
Immunoblot analysis with MeCP2 antibody revealed multiple MeCP2 immunoreactive bands at the same position as the fluorescent signals detected via SDS-PAGE and in gel fluorescence scanning. (**A**) Fluorescence pattern of total cell lysate from hMeCP2e1-RFP^+^ N2A neural cell line. (**B,C,D**) Fluorescence pattern, Ponceau staining and MeCP2 immunoblot of total cell lysate from hMeCP2e1-RFP^+^ SHSY5Y neural cell line. (**E,F,G**) Fluorescence pattern, Ponceau staining and MeCP2 immunoblot of total cell lysate from HEK293 and hMeCP2e1-RFP^+^ HEK293 cell lines. Protein ladder (M) and protein size markers (in kilodaltons) are indicated on the right of each panel.

Lastly, to demonstrate the specificity of multiple MeCP2 immunoreactive bands detected in hMeCP2e1-RFP expressing neural cell lines, and therefore, definitely exclude the cross-reactivity with similar epitopes on other proteins, we performed MeCP2e1-RFP detection via SDS-PAGE and in-gel fluorescence scanning (Fig 4). The scanning was performed on a Typhoon FLA 9500 scanner using 432 nm excitation laser and 610 BP40 emmision filter. After the fluorescence scan (Fig 4A, 4B and 4E), proteins in gels were transferred to nitrocellulose membranes and stained with Ponceau solution (Fig 4C and 4F). Immunoblot analysis with antibody against the C-terminal region of MeCP2 protein (H300, a.a.198-496) revealed multiple MeCP2 immunoreactive bands at the same position as the fluorescent signals (Fig 4D and 4G).

Our data clearly indicate that MeCP2 antibodies have no cross-reactivity with similar epitopes on others proteins.

### MeCP2 immunoreactive bands differences between wild-type and p.T158M MeCP2e1-RFP mutant neural expressing cells

Different MeCP2 mutations have been identified in individuals with Rett syndrome (RettBase: IRSF *MECP2* Variation Database; http://mecp2.chw.edu.au). One of the most common MECP2 mutations associated with Rett syndrome is p.T158M [21].

**Fig 5:**
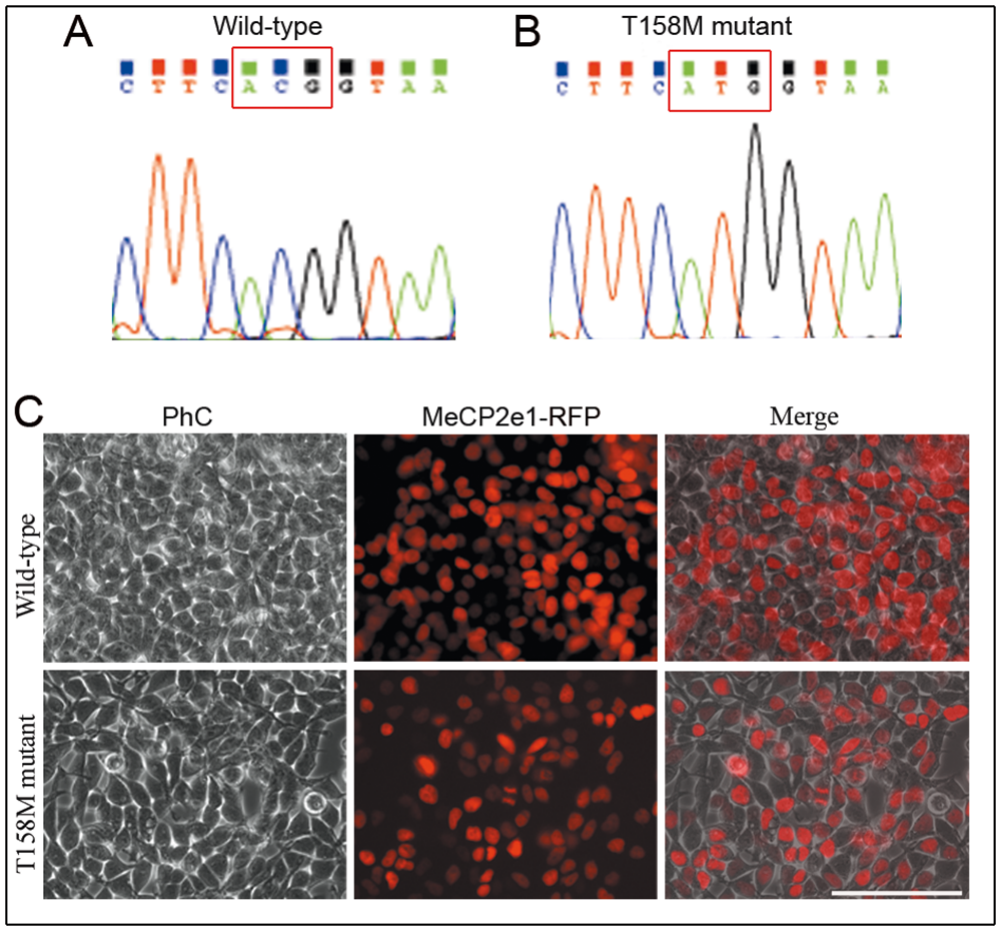
p.T158M MeCP2e1-RFP mutant expressing neural cell line. (**A**) Sequencing chromatogram of hMeCP2e1-RFP expression vector. Red box indicated codon ACG (threonine). (**B**) Single nucleotide mutation converting T158 to methionine (T158M). Sequencing chromatogram of mutant hMeCP2e1-RFP expression vector. Red box indicated codon ATG (methionine). (**C**) Photomicrographs show phase-contrast (PhC) and fluorescence images of wild-type and p.T158M hMeCP2e1-RFP^+^ mutant expressing neural cell line. Scale bar = 100μm.

With the intention of determining whether wild-type and hMeCP2 mutant neural cell lines differ in MeCP2 immunoreactive bands, we have generated p.T158M MeCP2e1-RFP mutant fusion protein (Fig 5A and 5B). HEK293 cell line was transfected with eukaryotic expression vector carrying mutated hMeCP2e1-RFP fusion protein (as described in Methods). Mutant hMeCP2e1-RFP^+^ expressing neural cell line, after months of continuous drug selection, rendered growing cultures in which most of cells were fluorescent under the microscope (Fig 5C). The fluorescence intensity in mutant cells is lower than in wild-type cells.

**Fig 6:**
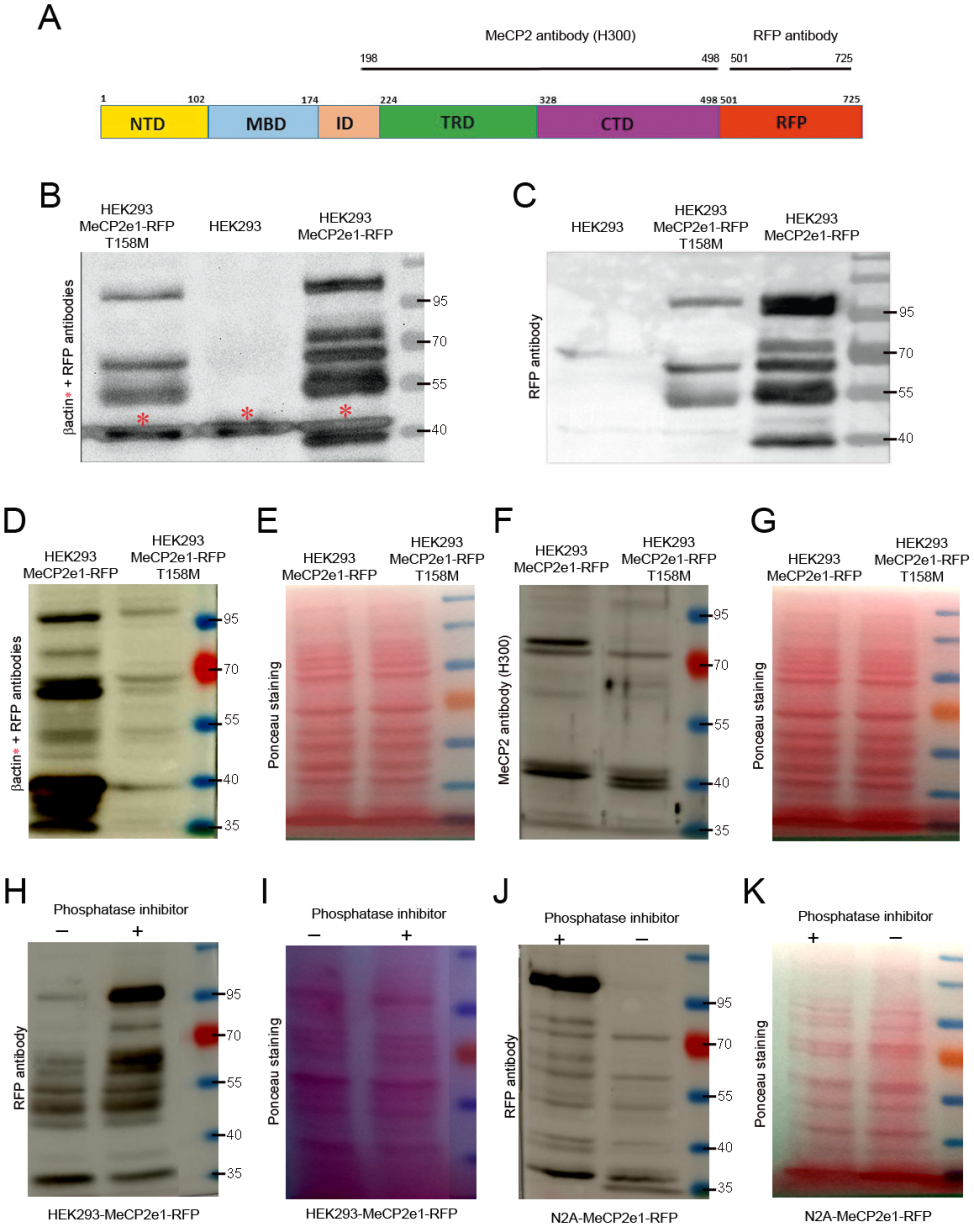
Multiple MeCP2 and RFP immunoreactive bands in p.T158M MeCP2e1-RFP mutant expressing neural cell line. (**A**) Diagram of the hMeCP2e1-RFP protein illustrating the position of the MeCP2 and RFP antibodies. (**B**) RFP immunoreactive bands in wild-type and p.T158M hMeCP2e1-RFP^+^ mutant expressing neural cell lines. Blots were also double-stained for β-actin, as a loading control. The asterisks marks β-actin bands. (**C**) Higher denaturing conditions did not affect the recognition of the RFP immunoreactive bands. (**D-G**) RFP and MeCP2 immunoreactive bands in wild-type and p.T158M hMeCP2e1-RFP^+^ mutant expressing neural cell lines. (**H-K**) Only one RFP immunoreactive band around 70kDa (faster migration band) was visible in hMeCP2e1-RFP^+^ HEK293 and N2A cell lines in the absence of phosphatase inhibitor. Blots were stained with Ponceau solution as a loading control. Protein size markers (in kilodaltons) are indicated on the right of each panel.

To assess hMeCP2-RFP expression at the protein level, immunoblot analysis with MeCP2 and RFP antibodies (Fig 6A) was carried out on total cell lysate from proliferating wild-type and mutant hMeCP2e1-RFP^+^ neural cell lines (Fig 5B and 5C). The MWa of RFP immunoreactive bands in wild-type hMeCP2e1-RFP transfected cells was around 95kDa, 70kDa (two bands), 55kDa and 40kDa, while in p.T158M MeCP2e1-RFP mutant transfected neural cells was around 95kDa, 70kDa and 55kDa. Staining with β-actin antibody (42kDa) was used as a loading control (Fig 6B). Higher denaturing conditions obtained by boiling the samples prior to electrophoresis did not affect the recognition of the RFP immunoreactive bands (Fig 6C).

The main difference between wild-type and p.T158M MeCP2e1-RFP mutant expressing neural cell lines was that the latter detected less RFP immunoreactive bands. Only one immunoreactive band around 70kDa (faster migration band) was visible in p.T158M MeCP2e1-RFP expressing cell line. Having repeated this experiment several times, always only one RFP immunoreactive band around 70kDa (faster migration band) was detected in mutant (T158M) hMeCP2e1-RFP^+^ cell line (Fig 6D and 6E). Staining with the C-terminal MeCP2 antibody, also only one MeCP2 immunoreactive band around 70kDa (faster migration band) was detected in mutant (T158M) hMeCP2e1-RFP^+^ cell line (Fig 6F and 6G). Blots were stained with Ponceau solution as a loading control.

Protein phosphorylation is an important reversible posttranscriptional modification that can modulate the function of MeCP2 protein via the addition of a phosphate group to serine, tyrosine or threonine residues [22]. Phosphorylation often causes proteins to migrate more slowly through acrylamide gels, therefore, the observed slower migration MeCP2 immunoreactive band around 70kDa it could be due to protein phosphorylation.

To check this, hMeCP2e1-RFP^+^ HEK293 and N2A neural cell lines protein extraction was carried out in the presence or absence of phosphatase inhibitor. Only one RFP immunoreactive band around 70kDa (faster migration band) was visible in hMeCP2e1-RFP^+^ HEK293 and N2A neural cell lines in the absence of phosphatase inhibitor (Fig 6H–6K). Blots were stained with Ponceau solution as a loading control.

hMeCP2-T158M mutation occurs at threonine 158, converting it to methionine. Our data suggest that threonine 158 could represent an important phosphorylation site potentially involved in protein function.

## Discussion

MeCP2 has been studied extensively and its functions have been expanded dramatically in the past two decades, which has resulted in the identification of pleotropic functional properties of this protein, however, the exact functions of MeCP2 protein is still far from clear [3,5,6,22–24]. At a molecular level, there exist contradictory data. MeCP2 protein is considered a single MeCP2 immunoreactive band around 75kDa by western-blot analysis but several previous reports have revealed the existence of multiple MeCP2 immunoreactive bands above and below the level where MeCP2 is expected [1,10–18]. Multiple MeCP2 immunoreactive bands have been interpreted in different ways. Some researchers suggest that multiple MeCP2 immunoreactive bands are unidentified proteins that cross-react with the MeCP2 antibody [11,12,15–17] or degradation product of MeCP2 [1,14], while others suggest that hMeCP2 post-transcriptional processing generates multiple molecular forms linked to cell signaling [10,18].

Our results clearly indicate that MeCP2 antibodies have no cross-reactivity with similar epitopes on others proteins, supporting the idea that MeCP2 may exist in multiple different molecular forms. We acknowledge that the definitive confirmation that MeCP2 may exist in multiple molecular forms will be provided only by direct amino acid sequencing of MeCP2 immunoreactive bands (currently under way) but several data suggest it.

Firstly, as noted before, previous reports have revealed the existence of multiple MeCP2 immunoreactive bands above and below the level where MeCP2 is expected [1,10–18].

Second, as note in the introduction, many MeCP2 antibodies available commercially against different epitopes of MeCP2 protein detected multiple bands at various molecular weights by western-blot analysis (Table 1).

Third, we observed the existence of multiple MeCP2 immunoreactive bands in several proliferating neural cell lines. Application of N- and C- terminal MeCP2 antibodies minimized concerns about nonspecific cross-reactivity. No large differences in the MWa of MeCP2 immunoreactive bands were noticed between our results, previous reports and MeCP2 antibodies available commercially against different epitopes of MeCP2 protein.

Fourth, no large differences in the MWa of multiple MeCP2 immunoreactive bands were detected between control neural cells and hMeCP2e1-RFP transfected neural cell lines. In addition, staining with RFP antibody, that minimized concerns about nonspecific cross-reactivity, produced blots with similar pattern. No large differences in the MWa of MeCP2 immunoreactive bands were noticed between different experiments, although the intensity of MeCP2 immunoreactive bands sometimes varied from one experiment to another.

Fifth, to demonstrate the specificity of multiple MeCP2 immunoreactive bands detected in hMeCP2e1-RFP expressing neural cell lines, and therefore, definitely exclude the cross-reactivity with similar epitopes on other proteins, we performed MeCP2e1-RFP protein detection via SDS-PAGE and in-gel fluorescence scanning. After the fluorescence scan, proteins in gels were transferred to nitrocellulose membranes for western blotting. The immunoblot with antibody against MeCP2 revealed multiple MeCP2 immunoreactive bands at the same position as the fluorescent signals.

Lastly, we found differences in the number of MeCP2 immunoreactive bands between wild-type and p.T158M MeCP2e1-RFP mutant expressing neural cell lines. Slower migration phosphorylated MeCP2 immunoreactive band around 70kDa disappeared in mutant pT158M neural cell lines.

One of the most common MECP2 mutations associated with Rett syndrome is p.T158M [21]. MeCP2 mutation p.T158M occurs at threonine 158, converting it to methionine. Our results suggest that this particular threonine could represent an important phosphorylation site potentially involved in protein function.

Multiple molecular forms of MeCP2 protein with different functional protein domain**s** and post-transcriptional modification may explain the functional complexity of the MeCP2 protein. However, futures studies investigating the amino acid composition of MeCP2 immunoreactive bands will be required to understand the function of these putative MeCP2 molecular forms. Although Rett syndrome phsyopathology represent one of the most frequent forms of severe intellectual disability in females, the molecular mechanisms through which different types of MeCP2 mutation lead to disruptions in proper brain function are not fully understood [3,5,6,22–24]. The lacking of phosphorylated MeCP2 in p.T158M mutant cells may represent a seminal data to stimulate deeper molecular studies trying understand how different conformations of MeCP2 molecular pattern regulates neuronal maturation.

Finally, since it has been proved the possibility to experimentally revert endophenotypic manifestations in adult mouse models of Rett syndrome [25], it is important to investigate whether the consecutive expression of MeCP2 pattern may explain the temporal pattern of functional phenotypes of this syndrome.

## Conclusions

In summary, our results clearly indicate that MeCP2 antibodies have no cross-reactivity with similar epitopes on others proteins, supporting the idea that MeCP2 may exist in multiple different molecular forms and that molecular pattern variations derived from altered post-transcriptional processing may underlay Rett syndrome physiophatology

Finally, one of the most common MECP2 mutations associated with Rett syndrome is p.T158M. hMeCP2-T158M mutation occurs at threonine 158, converting it to methionine. Our data suggest that threonine 158 could represent an important phosphorylation site potentially involved in protein function.
